# Resource diversity and provenance underpin spatial patterns in functional diversity across native and exotic species

**DOI:** 10.1002/ece3.3998

**Published:** 2018-04-02

**Authors:** Verónica Méndez, Jamie R. Wood, Simon J. Butler

**Affiliations:** ^1^ School of Biological Sciences University of East Anglia Norwich UK; ^2^ Landcare Research Lincoln New Zealand

**Keywords:** avian community, environmental filtering, functional diversity, New Zealand, resource provision, resource use, simultaneous autoregressive models

## Abstract

Functional diversity metrics are increasingly used to augment or replace taxonomic diversity metrics to deliver more mechanistic insights into community structure and function. Metrics used to describe landscape structure and characteristics share many of the same limitations as taxonomy‐based metrics, particularly their reliance on anthropogenically defined typologies with little consideration of structure, management, or function. However, the development of alternative metrics to describe landscape characteristics has been limited. Here, we extend the functional diversity framework to characterize landscapes based on the diversity of resources available across habitats present. We then examine the influence of resource diversity and provenance on the functional diversities of native and exotic avian communities in New Zealand. Invasive species are increasingly prevalent and considered a global threat to ecosystem function, but the characteristics of and interactions between sympatric native and exotic communities remain unresolved. Understanding their comparative responses to environmental change and the mechanisms underpinning them is of growing importance in predicting community dynamics and changing ecosystem function. We use (i) matrices of resource use (species) and resource availability (habitats) and (ii) occurrence data for 62 native and 25 exotic species and 19 native and 13 exotic habitats in 2015 10 × 10 km quadrats to examine the relationship between native and exotic avian and landscape functional diversity. The numbers of species in, and functional diversities of, native and exotic communities were positively related. Each community displayed evidence of environmental filtering, but it was significantly stronger for exotic species. Less environmental filtering occurred in landscapes providing a more diverse combination of resources, with resource provenance also an influential factor. Landscape functional diversity explained a greater proportion of variance in native and exotic community characteristics than the number of habitat types present. Resource diversity and provenance should be explicitly accounted for when characterizing landscape structure and change as they offer additional mechanistic understanding of the links between environmental filtering and community structure. Manipulating resource diversity through the design and implementation of management actions could prove a powerful tool for the delivery of conservation objectives, be they to protect native species, control exotic species, or maintain ecosystem service provision.

## 1 INTRODUCTION

Patterns of species richness and community structure are underpinned by complex interactions between broad‐scale factors relating to the abiotic environment and historical biogeography, and local scale responses to resource availability and species interactions (Montaña, Winemiller, & Sutton, 2013). Biological invasions, climate change, and land‐use modification are shifting the direction and relative strength of these environmental filters, with consequent detrimental impacts on global biodiversity (Bellard, Bertelsmeier, Leadley, Thuiller, & Courchamp, 2012; Cisneros, Fagan, & Willig, 2015; Karp et al., 2012; Sala et al., 2000). Understanding how and why communities assemble and disassemble in the face of such changes, and the consequences of changing community structure on ecosystem function and service delivery, is crucial if we are to mitigate their impacts (Bellard et al., 2012; Cavender‐Bares, Kozak, Fine, & Kembel, 2009).

Until recently, species richness and turnover have generally been used to characterize both patterns of biodiversity among sites and community responses to environmental change (Dreiss et al., 2015; Thuiller et al., 2014; Tscharntke et al., 2012). However, there is increasing acknowledgment that such taxonomy‐based metrics provide only a limited impression of community structure and dynamics and that adopting a trait‐based, functional view can potentially offer both greater resolution and more mechanistic insights (Birkhofer et al., 2017; Devictor et al., 2010; Flynn et al., 2009; Petchey & Gaston, 2006); environmental filtering constrains species composition by selecting species that are functionally adapted to the given environmental conditions (Knapp & Kühn, 2012) and functional traits rather than taxonomic identity determine contribution to ecosystem function and service provision (Gagic et al., 2015). Using these approaches, it has been shown that environmental filtering can reduce functional diversity within a community by restricting occurrence to species with more similar traits, while higher functional diversity is indicative of processes, such as competition or facilitation that limit similarity or promote dissimilarity between species (Birkhofer et al., 2017; Petchey & Gaston, 2006; Valiente‐Banuet & Verdú, 2007).

Augmenting and replacing taxonomy‐based metrics with measures of functional diversity can provide a new dimension to explorations of community structure and function (Mouillot, Graham, Villéger, Mason, & Bellwood, 2013; Si et al., 2017; Wright et al., 2006). It is therefore surprising that, despite these benefits, the philosophy and reasoning underpinning these developments have yet to be used to develop equivalent descriptors of environmental filters; in many cases, current descriptors share the same limitations of taxonomy‐based metrics that underpinned the move toward greater consideration of functional dimensions of biodiversity. For example, many studies of functional diversity still relate it to landscape characteristics defined in terms of the composition or configuration of specific, anthropogenically defined habitat types (Cisneros et al., 2015; Gámez‐Virués et al., 2015; Hogg & Daane, 2013; Petchey, Evans, Fishburn, & Gaston, 2007), without accounting for similarities and differences in resource provision between habitats. Thus, a landscape composed of habitats providing similar resource types can be characterized as equivalent to a landscape containing the same number of habitat types but providing a diverse range of resources, even though the former is likely to support a smaller and less functionally diverse community. Given that the availability and abundance of resources dictate species’ associations with particular habitats (Fahrig et al., 2011), adopting a framework that accounts for resource diversity across habitats when characterizing landscapes is likely to provide additional understanding of the links between environmental filtering and community structure (Fahrig et al., 2011; Perović et al., 2015).

Here, we extend established methods for quantifying species’ functional diversity to characterize landscapes based on the diversity of resources available across habitats. We then use this approach to explore spatial patterns in, and the relationship between, the functional diversities of native and exotic bird communities. Biological invasions are increasing in prevalence (Hogg & Daane, 2013), and invasive species are considered a global threat to biodiversity and community interactions (Hejda, Pyšek, & Jarošík, 2009; Sanders, Gotelli, Heller, & Gordon, 2003). Understanding the response of sympatric native and exotic communities to changes in the balance of local environmental filters is therefore of particular importance for predicting overall community dynamics and changing ecosystem function (Cleland et al., 2004).

Many observational studies have shown a generally positive correlation between the species richness of native and exotic communities, suggesting they respond in similar ways to extrinsic factors or environmental filters (Cleland et al., 2004; Levine, 2000). In both native and exotic communities, specialist species are likely to be more sensitive to changes in resource availability than generalists (Butler, Vickery, & Norris, 2007; Clavel, Julliard, & Devictor, 2010), while generalists should be more able to respond positively to the creation of new niches arising from environmental change and become established (Didham, Tylianakis, Hutchison, Ewers, & Gemmell, 2005; Warren et al., 2001). Generalist species tend to be less functionally diverse or distinct than specialist species (Clavel et al., 2010), so the contrasting fortunes of generalist “winners” and specialist “losers” (McKinney & Lockwood, 1999) in communities can lead to a process of functional homogenization (Clavel et al., 2010; Thuiller et al., 2014). However, the contrasting effects of climate and habitat change on native and exotic species (Hogg & Daane, 2013; Marini et al., 2012) and the growing presence of exotic species in most communities (Didham et al., 2005) suggest that exotics may be better at exploiting environmental shifts that alter the nature and strength of environmental filtering. Furthermore, there is conflicting evidence showing that functional uniqueness does not necessarily increase species’ sensitivity to environmental change (Buisson, Grenouillet, Villéger, Canal, & Laffaille, 2013; Thuiller et al., 2014). Thus, while it is clear that different functional types respond to environmental change in different ways and that a community's composition will dictate its response to such changes (Barbet‐Massin & Jetz, 2015; Cadotte, Carscadden, & Mirotchnick, 2011; Rader, Bartomeus, Tylianakis, & Laliberté, 2014), the mechanisms underpinning these processes remain poorly understood.

We apply our approach specifically to New Zealand terrestrial systems, testing (i) whether sympatric native and exotic bird communities experience equivalent levels of environmental filtering, (ii) if resource diversity and provenance (i.e., within and across native and exotic habitats) influence the functional diversity of each community and (iii) whether models based on a functional characterization of landscapes explain a greater proportion of the variance in avian community richness and functional diversity than habitat‐based models. We take resource use by each species as a proxy for underlying functional traits and focus specifically on those related to foraging and nesting behavior when quantifying functional diversity for two main reasons. Firstly, we wanted to generate directly comparable metrics for avian communities and landscapes by insuring that the effective dimensionality and bounds of the ‘resource space’ onto which species and habitats were mapped were the same; including morphological, physiological, or behavior traits to describe functional diversity (Moretti et al., 2017) would have prevented this as they could not be mirrored in equivalent resource provision by habitats. Secondly, it has previously been shown that the quantity and quality of resources associated with foraging and reproduction can be used to delineate species’ functional space and that the availability of functional space defined in this way predicts species’ responses to land‐use change (Butler & Norris, 2013; Butler et al., 2007; Wade et al., 2013); in effect, the landscape functional diversity metric presented here reflects the composite functional space available in each quadrat.

## 2 METHODS

### 2.1 Avian distribution and landscape composition

Since human settlement 750 years ago, large‐scale habitat loss and modification have occurred across New Zealand and approximately one‐quarter of its terrestrial native avifauna has gone extinct (Wood, 2013). Over the same time period, many bird species have been introduced and become widely established. We extracted bird presence data from the New Zealand Bird Atlas (Robertson, 2007), which covers data recorded in 3138 10 × 10 km quadrats between 1999 and 2004. Our analyses were based on the combined species’ lists from both full (recording all species) and partial (submitted with a note that only a subset of species had been recorded) surveys, with the total number of lists submitted for each quadrat during the survey period used as a proxy for survey effort in our analyses (see below). Exclusively marine or recently feral species were excluded, as were species restricted to offshore islands and migratory species that do not breed in New Zealand, resulting in a species pool of 87 (62 native and 25 exotic; Table S1). Landscape composition data, based on the presence of 32 land cover classes (hereafter habitats), were extracted for each quadrat from the New Zealand Land Cover database for summer 2001/2002 (Terralink 2004); quadrats containing more than 10% sea or with no species recorded were excluded. To allow the influence of resource provenance to be explored, each habitat was classified as native (19) or exotic (13) (Table S2); below, we refer to the native and exotic habitats within a quadrat as its native or exotic landscape.

### 2.2 Resource use

We constructed a resource use matrix for the 87 species by collating data on diet, foraging strata, and nest site location (Table S3). A binary response (i.e., used or not used) was recorded for each foraging strata (6 categories) and nest location (7 categories). Similarly, potential dietary items (25 categories) were included as separate resource types in the matrix, with the degree of importance in each species’ diet recorded from 0 to 3 (0 ‐ not recorded feeding on this; 1 ‐ rare or incidental dietary item; 2 ‐ minor dietary item; 3 ‐ important dietary item) for each.

### 2.3 Resource availability

Resource availability in each of the 32 habitat types was categorized using an equivalent matrix structure to that used to characterize species’ resource use (Table S3). A binary response (i.e., present or not present) was used to record the availability of each foraging strata and nesting location type in each habitat, while the relative abundance of each potential dietary item was scored between 0 and 3 (0—habitat does not offer any real potential for this item; 1—item only available at low quantity and/or quality in this habitat; 2—item available in intermediate quality and/or quantity in this habitat; 3—item available in high quality and/or quantity in this habitat). Data used to populate the resource use and resource availability matrices were independently compiled from published literature and local expert knowledge, and complete matrices are available in full from Wood, MacLeod, Gormley, Tompkins, and Butler (2016).

### 2.4 Calculating functional diversity

Using Petchey and Gaston's (2006) functional diversity (FD) metric, four measurements of functional diversity were generated per quadrat. Specifically, resource use by the native species recorded in a quadrat was used to calculate its native community FD; resource use by the exotic species recorded in a quadrat was used to calculate its exotic community FD; the resource availability in native habitats present in a quadrat was used to calculate its native landscape FD; and the resource availability in exotic habitats present in a quadrat was used to calculate its exotic landscape FD. We calculated these four FDs for 2015 quadrats in which at least two species and two habitat types were recorded. The species‐by‐resource use and habitat‐by‐resource availability matrices were converted into distance matrices using Gower's distance (Gower, 1971) and clustered to produce functional dendrograms using unweighted pair‐group method with arithmetic means (UPGMA) (Figure S1); the functional diversity of a given community or landscape in a quadrat was then calculated as the total length of branches required to connect species or habitats present (Petchey & Gaston, 2006).

### 2.5 Null‐model methods

As FD can only remain the same or increase with the addition of a new species into a community or habitat into a landscape, it is positively correlated with component (i.e., species or habitat) richness (Petchey & Gaston, 2002). For each quadrat and each of the four measurements of functional diversity, we therefore used a simulation approach to generate null distributions of expected FDs based on the number of components present. This allowed a direct comparison between communities or landscapes with different component richness. For example, holding native species richness in a quadrat constant, we randomly selected the equivalent number of native species from the native species pool (i.e., all 62 species) to calculate a null FD for the native community in that quadrat, with the probability of a species being selected proportional to the number of quadrats in which it was recorded to ensure rare species did not have a disproportional influence (Mendez et al., 2012; Thompson et al., 2010). This process was iterated 1,000 times to produce a distribution of expected native community FD. From this, we calculated a standardized FD (sFD) for the native community in that quadrat by subtracting the average expected FD from the observed FD and dividing by the standard deviation of expected FD (Gotelli & McCabe, 2002); negative sFD values indicate that functional diversity is lower than expected and that relatively higher levels of environmental filtering are operating. This was repeated for native and exotic communities and native and exotic landscapes in each quadrat, drawing from the appropriate species or habitat pool in each case; the probability of a habitat being selected was proportional to the number of quadrats in which it was recorded. We also calculated expected native and exotic FDs by drawing from island‐specific pools to reflect the restricted distribution of certain species and habitats to just the North or South Island. However, there was a very high correlation (*r* > .99) between these values and those based on drawing from the combined island pools, so only the latter are presented.

### 2.6 Statistical analyses

We used linear regression to investigate the relationships between avian community and landscape characteristics. Specifically, we examined and compared the influence of (i) the number of native and exotic habitat types present (hereafter termed habitat models) and (ii) the functional diversity (both FD and sFD) of native and exotic landscapes in a quadrat (hereafter termed functional diversity models) on the taxonomic and functional diversity (both FD and sFD) of native and exotic communities. Given that species richness increased with survey effort (data not shown), log (effort) was included as a covariate in all models with either species richness or community FD as the dependent variable, where effort was defined as the number of species lists submitted for a given quadrat in the Bird Atlas; this was not required for models with a community sFD measure as the dependent variable as this metric is independent of species richness. Model fit was assessed by visual inspection of residuals plotted against fitted values and quantile plots, with models of exotic species richness and exotic community FD consequently refitted using their squared form as the dependent variable to improve normality of error structure. Analysis of residuals using Moran's *I* (Legendre, 1993) revealed strong spatial autocorrelation in all models. We therefore repeated the analyses using simultaneous autoregressive (SAR) models to add a spatially dependent error term to the ordinary least squares models (Dormann et al., 2007), applying stepwise backwards elimination of nonsignificant covariates using likelihood ratio tests and 5% significance (Beale, Lennon, Yearsley, Brewer, & Elston, 2010). A neighborhood distance of 35 km, with each quadrat having up to 36 neighbors, was set following comparison of Akaike information criteria and Moran's *I* values at sequentially increasing neighborhood distances. We assessed the average contribution of each predictor to the variance in species richness and community functional diversity using hierarchical partitioning. First, we removed the spatial component from the fitted values from the SAR models and used the resultant values as a new response variables in the hierarchical partitioning procedure, using the metric ‘*lmg*’ in the R package *relaimpo* to assess the importance of predictors in explaining the variance that is not attributable to the random spatial component (Belmaker & Jetz, 2011; Grömping, 2006). Habitat and functional diversity models for each dependent variable (species richness, FD, and sFD for native and exotic avian communities) were compared using Akaike information criteria (AIC) of the simultaneous autoregressive models and *R* ^2^ values derived from the hierarchical partitioning, with comparisons of *R* ^2^ made between the combined explanatory power of all retained predictors except log (effort) in each model. All statistical analyses were performed using *spdep*,* ncf,* and *relaimpo* the R statistical program (R Development Core Team, 2014).

## 3 RESULTS

**Figure 1:**
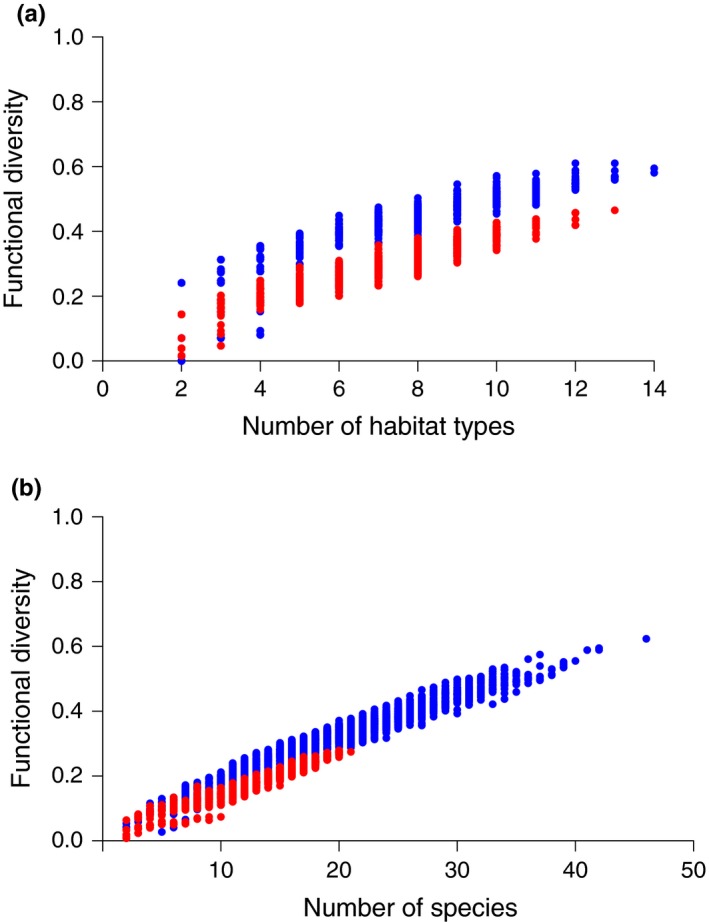
Relationship between functional diversity and (A) the number of habitats and (B) the number of species present in a quadrat. Red—exotic; blue—native

**Figure 2:**
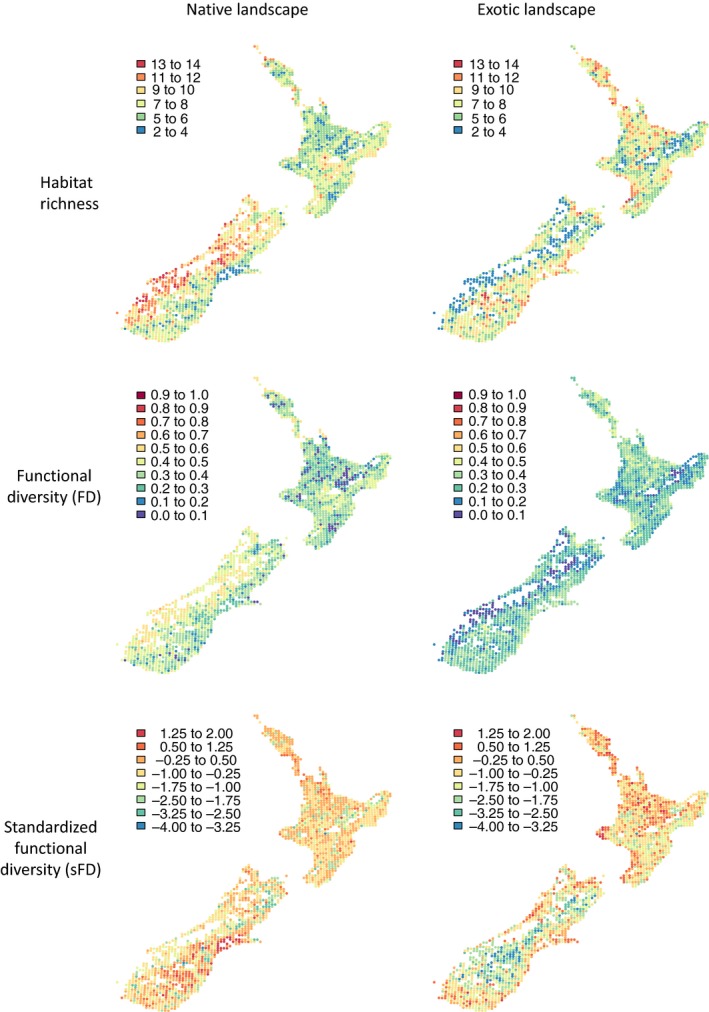
The number of habitats (first row), functional diversity (FD, second row), and standardized functional diversity (sFD, third row) of native (first column) and exotic (second column) landscapes in each 10 × 10 km quadrat

The number of native and exotic habitat types in a quadrat ranged from 2 to 14 (mean ± *SE* = 6.55 ± 0.05) and 2–13 (6.24 ± 0.05), respectively. The number of habitat types present in a landscape strongly influenced its functional diversity (Figure 1a), with native landscapes tending to be more functionally diverse than landscapes containing equivalent numbers of exotic habitats. There was little spatial congruence in the functional diversity of native and exotic landscapes (Figure 2), with a very weak, albeit statistically significant, negative correlation in values across quadrats (*r *=* *−.05, *p *<* *.05).

### 3.1 Taxonomic diversity

**Figure 3:**
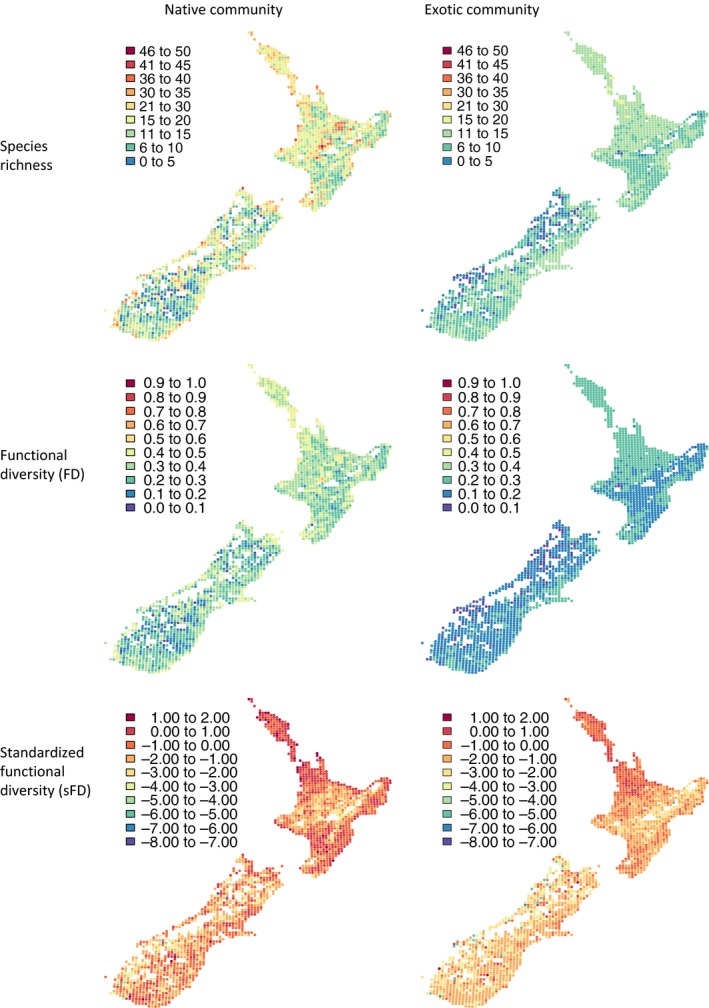
The number of species (first row), functional diversity (FD, second row), and standardized functional diversity (sFD, third row) of native (first column) and exotic (second column) communities in each 10 × 10 km quadrat

Native and exotic species richness per quadrat ranged from 2 to 46 (19.9 ± 0.16) and 2–21 (13.7 ± 0.08), respectively (Figure 3). There was a significant positive correlation between the richness of native and exotic communities in each quadrat (*r *=* *.56, *p *<* *.001).

The number of native species recorded in a quadrat was positively related to the number of native habitat types present (Table 1). Similarly, quadrats with more functionally diverse native landscapes supported a greater number of native species. However, for any given value of native landscape FD, native species richness was higher if observed native landscape FD was lower than expected given the number of habitat types present (Table 1). Neither the number of exotic habitat types present nor functional diversity of the exotic landscape influenced the number of native species recorded.

**Table 1:** Parameter estimates (β) and Z‐statistic from simultaneous autoregressive (SAR) models explaining the relationship between avian community and landscape characteristics

Avian		Landscape characteristics							
Community	Metric	Habitat model	β	Z	*R* ^2^ (%)	Functional Diversity model	β	Z	*R* ^2^ (%)
Native	SR	Log (Effort)	5.96	42.3^c^	55.3	Log (Effort)	5.91	41.7^c^	54.1
		Number native habitats	0.64	11.0^c^	3.4	FD native landscape	13.75	11.2^c^	3.9
						sFD native landscape	−0.55	−4.15^c^	.8
			AIC	11 644			AIC	11 639	
			Total *R* ^2^	3.4%			Total *R* ^2^	4.7%	
	FD	Log (Effort)	0.08	38.8^c^	51.1	Log (Effort)	0.08	38.7^c^	49.6
		Number native habitats	0.007	9.86^c^	2.5	FD native landscape	0.16	10.5^c^	3.4
		Number exotic habitats	0.003	5.01^c^	8.3	sFD native landscape	−0.004	−2.49^a^	1.0
						FD exotic landscape	0.07	3.70^c^	8.2
			AIC	−5960			AIC	−5969.1	
			Total *R* ^2^	10.8%			Total *R* ^2^	12.6%	
	sFD	Number exotic habitats	0.12	11.9^c^	10.4	sFD native landscape	0.09	3.75^c^	2.0
						FD exotic landscape	3.57	11.9^c^	10.1
						sFD exotic landscape	−0.10	−4.39^c^	.7
			AIC	5264.6			AIC	5241.1	
			Total *R* ^2^	10.4%			Total *R* ^2^	12.8%	
Exotic	SR^d^	Log (Effort)	43.0	26.5^c^	35.9	Log (Effort)	42.70	26.4^c^	33.3
		Number exotic habitats	8.55	15.3^c^	20.8	FD native landscape	−25.87	−1.99^a^	.4
						sFD native landscape	4.33	3.13^b^	2.1
						FD exotic landscape	278.61	16.5^c^	20.9
						sFD exotic landscape	−9.75	−8.09^c^	2.1
			AIC	21 109			AIC	21 069	
			Total *R* ^2^	20.8%			Total *R* ^2^	25.5%	
	FD^d^	Log (Effort)	0.006	24.0^c^	32.9	Log (Effort)	0.007	23.7^c^	30.4
		Number exotic habitats	0.001	16.1^c^	22.0	sFD native landscape	<0.001	2.58^b^	2.0
						FD exotic landscape	0.05	16.5^c^	21.3
						sFD exotic landscape	−0.001	−6.84^c^	2.1
			AIC	−13 707			AIC	−13 728	
			Total *R* ^2^	22.0%			Total *R* ^2^	25.4%	
	sFD	Number exotic habitats	0.06	7.80^c^	5.6	FD exotic landscape	1.69	7.45^c^	5.7
			AIC	4568.4			AIC	4573.8	
			Total *R* ^2^	5.6%			Total *R* ^2^	5.7%	

Avian community metrics are species richness (SR), functional diversity (FD) and standardized FD (sFD). Akaike information criterion (AIC) relates to SAR models while *R* ^2^ values are derived from hierarchical partitioning of the total variance not attributable to the random spatial component. We present the relative contribution of each predictor (*R* ^2^) and the combined explanatory power of all retained predictors except effort (total *R* ^2^) to allow comparison of habitat and functional diversity model types.

a *p *<* *.05.

b *p *<* *.01.

c *p *<* *.001.

d Entered in squared form.

The number of exotic species recorded in a quadrat was positively related to the number of exotic habitat types present but not the number of native habitat types (Table 1). The functional diversity of the exotic landscape also had a significant positive influence on exotic species richness, while quadrats with more functionally diverse native landscapes held fewer exotic species. For any given value of exotic landscape FD, exotic species richness was higher if the observed exotic landscape FD was lower than expected given the number of habitat types present while the opposite was true for native landscape FD (Table 1).

### 3.2 Functional diversity

**Figure 4:**
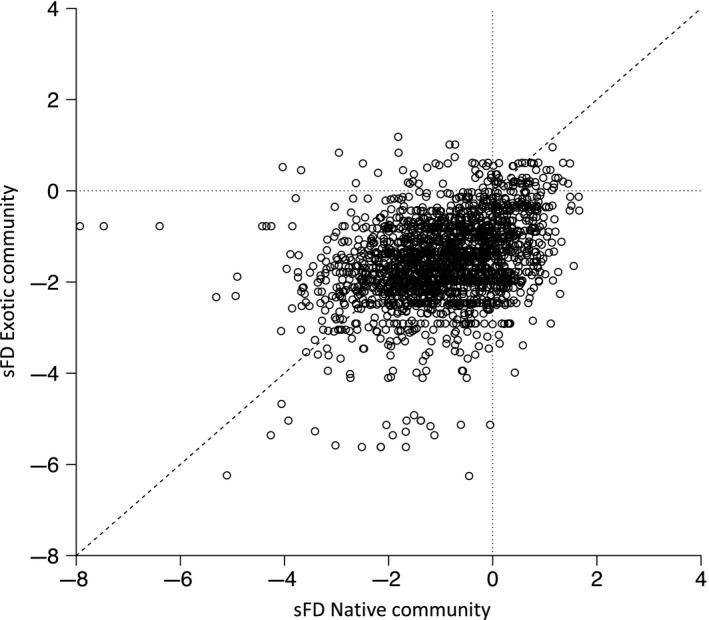
The standardized functional diversity (sFD) of the native community against the standardized functional diversity of the exotic community in each 10 × 10 km quadrat. Dashed line represents 1:1 relationship; horizontal and vertical dotted lines indicate the origin

For both native and exotic communities, functional diversity was strongly related to species richness and there was again little evidence of functional redundancy (Figure 1b). In both native and exotic communities, observed functional diversity was significantly lower than expected (one sample *t* test of sFD against an expected value of 0: native community *t *=* *−38.5, *p *<* *.001; exotic community *t *=* *−73.5, *p *<* *.001). There was a significant positive correlation between the functional diversities of sympatric native and exotic communities (FD: *r *=* *.61, *p *<* *.001), even when species richness was accounted for (sFD: *r *=* *.33, *p *<* *.001), but the functional diversities of exotic communities were significantly further below expected values than those of native communities (paired *t* test: *t *=* *23.0, *p *<* *.001; Figure 4).

The functional diversity of native communities was higher in landscapes containing more native and exotic habitat types (Table 1). Furthermore, landscapes with more exotic habitats supported native communities that had closer to expected levels of functional diversity, indicating lower levels of environmental filtering. Native community FD was positively related to both native and exotic landscape FDs but was lower if the observed FD of the native landscape was closer to expected levels given the number of native habitat types present (Table 1). Native community FD was also closer to expected levels if the exotic landscape functional diversity was higher or the native landscape functional diversity was closer to expected levels given the number of native habitat types present (Table 1).

The exotic communities recorded in landscapes containing more exotic habitat types were both more functionally diverse and had functional diversities closer to expected values (Table 1). However, the number of native habitats present in a quadrat did not influence exotic community FD. Exotic community FD increased with increasing exotic landscape FD and was closer to expected levels in quadrats with higher exotic landscape FD. Native landscapes with functional diversities closer to expected levels given the number of habitat types present also supported more functionally diverse exotic communities. For any given value of exotic landscape FD, exotic community FD was higher if the exotic landscape FD was further below expected levels given the number of exotic habitat types present (Table 1).

### 3.3 Comparison of Functional Diversity and Habitat models

Functional diversity models performed better than habitat models in explaining variance in both native and exotic species richness. For native species richness, neither model type explained a substantial component of the variance in numbers recorded but it was higher for the functional diversity model (4.7% versus 3.4%), which also had a lower AIC (Table 1). Both habitat and functional diversity models explained a much greater proportion of the variance in exotic species richness (20.8% and 25.5%, respectively) and again the functional diversity model had the lower AIC (Table 1).

Functional diversity models performed better than habitat models in explaining variance between quadrats in native community FD (12.6% vs. 10.8%), native community sFD (12.8% vs. 10.4%), and exotic community FD (25.4% vs. 22.0%). In each case, the functional diversity model also had a substantially lower AIC than its equivalent habitat model (Table 1). The performance of the two model types in explaining exotic community sFD was more equivocal, with the habitat model having a lower AIC (ΔAIC = 5.4) but the functional diversity model explaining a marginally higher proportion of variance (5.7% vs. 5.6%).

Interestingly, hierarchical partitioning showed that, for both functional diversity and habitat models, effort explained about half the variance in native species richness and community FD but only about one‐third of the variance in exotic species richness and community FD. In line with this, the relative explanatory power of landscape characteristics (either number of habitats or functional diversity) was higher for models of exotic species richness and community FD than for the equivalent model for native species (Table 1). Finally, where both native and exotic landscape functional diversity characteristics were retained in a model, variables describing the exotic landscape explained a greater proportion of variance than those describing the native landscape (Table 1).

## 4 DISCUSSION

We show that the number of species in, and functional diversity of, avian communities are influenced by the functional diversity of landscapes, with native and exotic species responding to both the diversity and provenance of resources available. We also reveal that, while both native and exotic communities display evidence of environmental filtering, these effects are significantly stronger for exotic species. With the functional diversity models outperforming the habitat models in five of the six comparisons made here, our analyses suggest that adopting a more functional, resource diversity‐based characterization of landscapes may provide additional insight into species richness and community functional diversity than approaches using metrics underpinned by anthropogenically defined habitat types.

Increased habitat heterogeneity is widely acknowledged as having a positive influence on animal species richness and functional diversity (Cadotte et al., 2011; Flynn et al., 2009; Tews et al., 2004). However, the metrics used to describe and quantify compositional and configurational heterogeneity (Bohning‐Gaese, 1997; Devictor, Julliard, Couvet, Lee, & Jiguet, 2007; Morelli et al., 2013) are constrained by their reliance on anthropogenically defined habitat types and allow little ecological consideration of structure, management, or function. This limitation is akin to that associated with using species richness metrics to describe community composition which underpinned the movement toward metrics of functional rather than taxonomic diversity (Wright et al., 2006). Our results support our argument that extending functional approaches to landscape characterization can offer similar additional insights to those gained when applying them to communities. Firstly, functional diversity models appear to identify additional drivers of avian community functional diversity. For example, habitat models of native community sFD and exotic species richness and community FD only retained exotic habitat richness as a predictor. However, functional diversity models of the same community characteristics indicated that both native and exotic landscape characteristics are influential. Secondly, although landscape FD is positively correlated with habitat richness (Figure 1a), the greater explanatory power of functional diversity models than habitat models, and the retention of a measure of both landscape FD and sFD in all but one functional diversity model, suggests that the relationship between community characteristics and landscape composition goes beyond established habitat heterogeneity effects, with resource diversity and provenance significantly influencing patterns of community assemblage (Josefsson, Berg, Hiron, Pärt, & Eggers, 2017).

Across New Zealand, avian functional diversity was lower than expected; among both the native and exotic communities, co‐occurring species were more similar in functional traits than expected by chance. This suggests that environmental filtering is operating and outweighs any influence of processes limiting similarity or promoting dissimilarity that would otherwise result in higher than expected functional diversity (Edwards, Edwards, Hamer, & Davies, 2013; Mendez et al., 2012; Petchey et al., 2007). That community functional diversity was closer to expected levels in more functionally diverse landscapes implies that limited resource diversity may be a strong contributor to environmental filtering. Species can only occur in landscapes that provide appropriate and sufficient functional space (Fahrig et al., 2011), so landscapes providing a limited range of similar resources (i.e., with low functional diversity) will only support a community of species that share functional space requirements and hence are functionally similar. It is important to note that it could be argued that the positive relationship between avian and landscape functional diversity is driven by changes in the strength of processes limiting similarity or promoting dissimilarity rather than environmental filtering. We believe the latter explanation is more plausible (Barnagaud, Barbaro, Papaïx, Deconchat, & Brockerhoff, 2014), with greater resource diversity broadening the composite functional space available and weakening environmental filtering rather than increasing levels of competition within communities. However, it is not possible to fully differentiate between these processes and this requires further exploration. The explanatory power of each simultaneous autoregressive model was high (Nagelkerke pseudo‐*R* ^2^ > .6 for all species richness and community FD models and >0.3 for both community sFD models; data not shown), but the majority of this was attributable to the random spatial component and/or survey effort. While highly significant, the explanatory power of variables relating specifically to habitat richness or landscape functional diversity was somewhat weaker, falling between 3.4% and 25.4% depending on model type and community characteristic. Other factors, such as configurational heterogeneity (Fahrig et al., 2011), are likely to influence the function of landscape characteristics as an environmental filter, and it in itself is just one of a suite of filters potentially operating, so this level of explanatory power is not necessarily surprising and is in line with that reported for similar analyses exploring the influence of landscape structure on avian species presence/absence (Radford & Bennett, 2007) and functional diversity (Petchey et al., 2007).

The richness and functional diversity of native and exotic communities, and the degree of environmental filtering they exhibited, were positively correlated, suggesting that their direction of response to environmental factors is similar (Maitner, Rudgers, Dunham, & Whitney, 2012). However, contrary to Knapp and Kühn (2012), who stated that environmental filtering should depend solely on species characteristics, not on their native/non‐native status, our results suggest that the composition of exotic communities is more constrained than that of native communities; for any given landscape, the functional diversity of the exotic community present tended to be further below expectation than that of the native community. This could reflect a reduced ability of exotic species to exploit available resources, either directly or because they are outcompeted for them by native species (Cleland et al., 2004), a greater sensitivity of exotic species to other environmental factors such as climatic conditions that can also contribute to filtering (Marini et al., 2012) or a greater influence of factors that either limit similarity or promote dissimilarity acting on native species (Barnagaud et al., 2014). Again, it is not possible to distinguish between these mechanisms here but all potentially reflect differences in the relative period of evolutionary adaptation to local conditions experienced by native and exotic species. That exotic community characteristics were better explained by landscape characteristics than were native community characteristics and that exotic landscapes tended to contribute more to model fit than native landscape characteristics are interesting and unexpected patterns. It may be that these results are also indicative of differences in the relative influence of other environmental filters on native and exotic communities or of more nuanced responses to landscape characteristics by each community. Certainly, our results suggest that the species richness and both the absolute and relative functional diversity of native and exotic communities are influenced by the absolute and relative functional diversity of landscapes and that the direction of these relationships can be influenced by whether resources are provided by native or exotic habitats. These results demonstrate the importance of considering provenance as well as abundance when considering the contribution of resource availability to environmental filtering (Case, 1996; Sol, Bartomeus, & Griffin, 2012), they require additional exploration and further discussion here would be purely speculative.

It is important to acknowledge that, due to the nature of the data available, our analyses are based on temporally static measures of functional diversity that do not incorporate abundance; while data on the area under each habitat type within each quadrat were available, count data were not recorded in the Bird Atlas dataset. This means our results do not consider the functional evenness or divergence components of either community or landscape functional diversity (Mouchet, Villéger, Mason, & Mouillot, 2010). Incorporating abundance, in terms of both individuals of each species and area under each habitat, into analyses could provide insight into important additional aspects of the mechanisms underpinning the role of resource diversity in environmental filtering and community composition (Mouchet et al., 2010). However, in New Zealand, predation limits many avian populations and abundance data are likely to be strongly influenced by the size and structure of the predator community (Innes, Kelly, Overton, & Gillies, 2010). Incorporating abundance data into metrics of functional diversity could therefore potentially mask important relationships between community structure and resource diversity, and they would need to be interpreted with caution.

## 5 CONCLUSION

Accounting for changes in resource provision is crucial to understanding the impacts of environmental change (Butler et al., 2007; Fahrig et al., 2011). In the same way that function‐based metrics have been developed to augment or replace taxonomy‐based metrics when describing community structure and dynamics, we suggest equivalent advances in the metrics used to describe landscapes and landscape change are required. Indeed, we would recommend that future studies of environmental change on community structure should explicitly account for changes in resource availability and diversity when exploring taxonomic and functional responses so as to develop a more mechanistic understanding of any relationship (Sullivan, Davies, Mossman, & Franco, 2015). More broadly, our findings suggest that conservation management, whether in terms of the protection of native species, control of exotic species, or maintenance of ecosystem function, could benefit from consideration of both resource availability and diversity (Hogg & Daane, 2013). Given that native and exotic communities respond differently to resource diversity and provenance, the benefits and costs of habitat restoration or land‐use change will be context dependent (Butler & Norris, 2013). In the same way that exotic species can maintain or enhance community functional diversity and ecosystem service delivery in the face of native species declines (García, Martínez, Stouffer, & Tylianakis, 2014), so exotic habitats could potentially be used to replace or supplement the resources provided by native habitats and their conservation value should not be dismissed simply because of their status (Martínez‐Abraín & Jiménez, 2016; Schlaepfer, Sax, & Olden, 2011). Manipulating resource diversity through the design and implementation of management actions could prove a powerful tool for the delivery of conservation objectives.

## AUTHOR CONTRIBUTIONS

SJB conceived the study and led the writing of the manuscript. JRW constructed the resource use and availability matrices, and VM led the data analyses. All authors contributed critically to the drafts and gave final approval for publication.
