# Supplementary material for: Resource diversity and provenance underpin spatial patterns in functional diversity across native and exotic species
Source: Ecol Evol. 2018 Apr 2;8(9):4409–21. doi: 10.1002/ece3.3998 (PMC5938469; doi:10.1002/ece3.3998)
Supplement: Supplementary file 1 [file ECE3-8-4409-s001.docx]

Figure S1: Functional dendrogram of a) 87 species and b) 32 habitats included in analyses. Dendrograms produced by hierarchical clustering using UPGMA algorithm of distance matrices calculated from resource provision and resource use respectively. Vertical distance represents separation in trait space, horizontal distance is for clarity. For species and habitat codes see Table S1 and Table S2 respectively.

(a)

(b)
