# Supplementary material for: Resource diversity and provenance underpin spatial patterns in functional diversity across native and exotic species
Source: Ecol Evol. 2018 Apr 2;8(9):4409–21. doi: 10.1002/ece3.3998 (PMC5938469; doi:10.1002/ece3.3998)
Supplement: Supplementary file 2 [file ECE3-8-4409-s002.docx]

Table S1. Definition of species codes used in Figure S1. Taxonomy based on: Gill, B., Bell, B., Chambers, G., Medway, D., Palma, R., Scofield, R. *et al.* (2010). *Checklist of the birds of New Zealand*. Wellington: Ornithological Society of New Zealand.

| Code | Common name | Scientific name | Classification |
| --- | --- | --- | --- |
| belbir | Bellbird | Anthornis melanura | Native |
| bitaus | Australasian bittern | Botaurus poiciloptilus | Native |
| blabir | Blackbird | Turdus merula | Exotic |
| brocre | Brown creeper | Mohoua novaeseelandiae | Native |
| buncir | Cirl bunting | Emberiza cirlus | Exotic |
| chafin | Chaffinch | Fringilla coelebs | Exotic |
| chukor | Chukor | Alectoris chukar | Exotic |
| cooaus | Australian coot | Fulica atra | Native |
| cramar | Ballion's crake | Porzana pusilla | Native |
| craspo | Spotless crake | Porzana tabuensis | Native |
| cuclon | Long-tailed cuckoo | Eudynamys taitensis | Native |
| cucshi | Shining cuckoo | Chrysococcyx lucidus | Native |
| dabchi | New Zealand dabchick | Poliocephalus rufopectus | Native |
| dotban | Banded dotterel | Charadrius bicinctus | Native |
| dotbla | Black-fronted dotterel | Elseyornis melanops | Native |
| dotnz | New Zealand dotterel | Charadrius obscurus | Native |
| dovbar | Barbary dove | Streptopelia risoria | Exotic |
| dovspo | Spotted dove | Streptopelia chinensis | Exotic |
| ducgre | Grey duck | Anas superciliosa | Native |
| falcon | New Zealand falcon | Falco novaeseelandiae | Native |
| fantai | New Zealand fantail | Rhipidura fulginosa | Native |
| ferbir | Fernbird | Bowdleria punctata | Native |
| golfin | European goldfinch | Carduelis carduelis | Exotic |
| goocan | Canada goose | Branta canadensis | Exotic |
| grefin | European greenfinch | Carduelis chloris | Exotic |
| gulbba | Southern black-backed gull | Larus dominicanus dominicanus | Native |
| gulbbi | Black-billed gull | Larus bulleri | Native |
| harrie | Swamp harrier | Circus approximans | Native |
| herree | Reef heron | Egretta sacra | Native |
| herwfa | White-faced heron | Egretta novaehollandiae | Native |
| herwhi | White heron | Ardea modesta | Native |
| kaka | Kaka | Nestor meridionalis | Native |
| kea | Kea | Nestor notabilis | Native |
| kinfis | New Zealand kingfisher | Todiramphus sanctus vagans | Native |
| kiwbro | South Island brown kiwi | Apteryx australis | Native |
| kiwgre | Great spotted kiwi | Apteryx haastii | Native |
| kokako | North Island kokako | Callaeas wilsoni | Native |
| magpie | Australian magpie | Gymnorhina tibicen | Exotic |
| mallar | Mallard | Anas platyrhynchos | Exotic |
| morpor | Morepork | Ninox novaeseelandiae | Native |
| myna | Common myna | Acridotheres tristis | Exotic |
| owllit | Little owl | Athene noctua | Exotic |

Table S1 continued.

| Code | Common name | Scientific name | | Classification |
| --- | --- | --- | --- | --- |
| oyssip | South Island pied oystercatcher | | Haematopus finschi | Native |
| parora | Orange-fronted parakeet | | Cyanoramphus malherbi | Native |
| parred | Red-crowned parakeet | Cyanoramphus novaezelandiae novaezelandiae | | Native |
| paryel | Yellow-crowned parakeet | Cyanoramphus auriceps | | Native |
| pheasa | Common pheasant | Phasianus colchicus | | Exotic |
| pignz | New Zealand pigeon | Hemiphaga novaeseelandiae | | Native |
| pigroc | Rock pigeon | Columba livia | | Exotic |
| pipit | New Zealand pipit | Anthus novaeseelandiae | | Native |
| plospu | Spur-winged plover | Vanellus miles novaehollandiae | | Native |
| pukeko | Pukeko | Porphyrio melanotus melanotus | | Native |
| quabro | Australian brown quail | Coturnix ypsilophora australis | | Exotic |
| quacal | California quail | Callipepla californica brunnescens | | Exotic |
| raiban | Banded rail | Gallirallus phillippensis | | Native |
| redpol | Common redpoll | Carduelis flammea | | Exotic |
| rifman | Rifleman | Acanthisitta chloris | | Native |
| robnz | South Island robin | Petroica australis | | Native |
| roseas | Eastern rosella | Platycercus eximius | | Exotic |
| scanz | New Zealand scaup | Aythya novaeseelandiae | | Native |
| shabl | Black shag | Phalacrocorax carbo novaehollandiae | | Native |
| shalbl | Little black shag | Phalacrocorax sulcirostris | | Native |
| shalit | Little shag | Phalacrocorax melanoleucos brevirostris | | Native |
| shapie | Pied shag | Phalacrocorax varius varius | | Native |
| shepar | Paradise shelduck | Tadorna variegata | | Native |
| shonz | Australasian shoveler | Anas rhynchotis | | Native |
| sileye | Silvereye | Zosterops lateralis | | Native |
| skylark | Eurasian skylark | Alauda arvensis | | Exotic |
| spahed | Dunnock | Prunella modularis | | Exotic |
| spahou | House sparrow | Passer domesticus | | Exotic |
| starli | Common starling | Sturnus vulgaris | | Exotic |
| stipie | Pied stilt | Himantopus himantopus | | Native |
| swabla | Black swan | Cygnus atratus | | Native |
| swawel | Welcome swallow | Hirundo neoxena | | Native |
| teagre | Grey teal | Anas gracilis | | Native |
| terbla | Black-fronted tern | Childonias albostriatus | | Native |
| tercas | Caspian tern | Hydroprogne caspia | | Native |
| thrson | Song thrush | Turdus philomelos | | Exotic |
| tomtit | Tomtit | Petroica macrocephala | | Native |
| tui | Tui | Prosthemadera novaeseelandiae | | Native |
| wargre | Grey warbler | Gerygone igata | | Native |
| weka | Weka | Gallirallus australis | | Native |
| whihea | Whitehead | Mohoua albicilla | | Native |
| wreroc | Rock wren | Xenicus gilviventris | | Native |
| wrybil | Wrybill | Anarhynchus frontalis | | Native |

Table S1 continued.

| Code | Common name | Scientific name | Classification |
| --- | --- | --- | --- |
| yelham | Yellowhammer | Emberiza citrinella | Exotic |
| yelhea | Yellowhead | Mohoua ochrocephala | Native |

Table S2. Definition of habitat codes used in Figure S1. Note HAB 32, Transport Infrastructure, was excluded from analyses.

| Habitat Code | Description | Classification |
| --- | --- | --- |
| HAB1 | Alpine Grass/Herbfield | Native |
| HAB2 | Broadleaved Indigenous Hardwoods | Native |
| HAB3 | Built-up Area | Exotic |
| HAB4 | Deciduous Hardwoods | Exotic |
| HAB5 | Depleted Grassland | Exotic |
| HAB6 | Estuarine Open Water | Native |
| HAB7 | Exotic Forest | Exotic |
| HAB8 | Fernland | Native |
| HAB9 | Flaxland | Native |
| HAB10 | Forest - Harvested | Exotic |
| HAB11 | Gorse/Broom | Exotic |
| HAB12 | Gravel/Rock | Native |
| HAB13 | Herbaceous Freshwater Vegetation | Native |
| HAB14 | Herbaceous Saline Vegetation | Native |
| HAB15 | High Producing Exotic Grassland | Exotic |
| HAB16 | Indigenous Forest | Native |
| HAB17 | Lake/Pond | Native |
| HAB18 | Landslide | Native |
| HAB19 | Low Producing Grassland | Exotic |
| HAB20 | Mangrove | Native |
| HAB21 | Manuka/Kanuka | Native |
| HAB22 | Matagouri/Grey Scrub | Native |
| HAB23 | Mixed Exotic Shrubland | Exotic |
| HAB24 | Orchard, Vineyard & Other Perennial Crops | Exotic |
| HAB25 | Permanent Snow/Ice | Native |
| HAB26 | River | Native |
| HAB27 | Sand/Gravel | Native |
| HAB28 | Short-rotation Cropland | Exotic |
| HAB29 | Sub Alpine Shrubland | Native |
| HAB30 | Surface Mine/Dump | Exotic |
| HAB31 | Tall Tussock Grassland | Native |
| HAB33 | Urban Parkland/Open Space | Exotic |

Table S3. Resource use (species) and resource availability (habitat) categories recorded in trait matrices from which data were extracted (Wood, et al. 2016).

| Resource type | Category |
| --- | --- |
| Nest location* | Floating |
|  | Burrow |
|  | Ground |
|  | Shrub |
|  | Tree |
|  | Cliff/Crevice |
|  | Man-made structure |
|  |  |
| Foraging strata* | Aquatic |
|  | Ground |
|  | Shrub |
|  | Sub-canopy |
|  | Canopy |
|  | Aerial |
|  |  |
| Dietary item** | Marine fish |
|  | Marine cephalopod |
|  | Marine crustacean |
|  | Marine other mollusca |
|  | Freshwater vegetation |
|  | Freshwater crayfish |
|  | Freshwater invertebrates |
|  | Freshwater fish |
|  | Carrion/Scavenger |
|  | Frog/Reptile |
|  | Bird eggs/small mammal |
|  | Soil invertebrate |
|  | Ground invertebrate |
|  | Flying invertebrate |
|  | Roots/rhizomes |
|  | Seeds |
|  | Pollen |
|  | Grass |
|  | Tree/shrub leaves |
|  | Stems/Shoots |
|  | Flower buds |
|  | Vegetable crops |
|  | Fruit |
|  | Bark/twigs |
|  | Nectar/sap/honeydew |

*Binary record of use/non-use (species) or availability/non-availability (habitat)

** Resource use: 0 - not recorded feeding on this; 1 - rare or incidental dietary item; 2 - minor dietary item; 3 - important dietary item; Resource availability: 0 - habitat does not offer any real potential for this item; 1 – item only available at low quantity and/or quality in this habitat; 2 – item available in intermediate quality and/or quantity in this habitat; 3 - item available in high quality and/or quantity in this habitat

Wood, J. R., et al. 2016. Ecological requirements of New Zealand birds. - In: Landcare Research NZ DataStore; <http://dx.doi.org/10.7931/J27D2S27>.
